# Biomass Allocation Responses to Root Interactions in Wheat Cultivars Support Predictions of Crop Evolutionary Ecology Theory

**DOI:** 10.3389/fpls.2022.858636

**Published:** 2022-03-23

**Authors:** Yong-He Zhu, Jacob Weiner, Yi Jin, Ming-Xi Yu, Feng-Min Li

**Affiliations:** ^1^Jiangsu Collaborative Innovation Center for Modern Crop Production, College of Agriculture, Nanjing Agricultural University, Nanjing, China; ^2^Department of Plant and Environmental Sciences, University of Copenhagen, Frederiksberg, Denmark; ^3^Key Laboratory of Vegetation Restoration and Management of Degraded Ecosystems, South China Botanical Garden, Chinese Academy of Sciences, Guangzhou, China; ^4^State Key Laboratory of Grassland Agroecosystems, Institute of Arid Agroecology, School of Life Sciences, Lanzhou University, Lanzhou, China

**Keywords:** belowground competition, crop ecology, root growth, root interactions, root proliferation, tragedy of the commons

## Abstract

The goal of agriculture is to optimize the population yield, but natural selection has produced active competition among plants, which decreases population performance. Therefore, cultivar breeding should be based on group selection, increasing yield by weakening individual competitive responses. We hypothesize that this has occurred inadvertently to some degree, so modern cultivars have weakened competitive traits and responses, such as reduced root proliferation in response to neighboring roots. We conducted a field experiment with eight cultivars of spring wheat that have been released over the last hundred years, which we grew at two densities. Two contrasting wheat cultivars, a landrace and a modern cultivar, were used in a second field experiment on competition within and between the two cultivars to quantify their competitiveness. Finally, a greenhouse experiment was conducted with these two cultivars gown (a) in mixture and monoculture, (b) at four densities, (c) two watering levels, and (d) with permeable vs. non-permeable soil dividers, to study root proliferation responses to competition. Results of field experiment 1 showed that the population aboveground biomass (AGB) had increased, while belowground biomass had decreased over the course of breeding, so that the root to shoot ratio (R/S) was negatively correlated with the release year of the cultivar. The landrace had stronger competitiveness than the modern cultivar in the field experiment 2. There was clear evidence of root proliferation and a resultant reduction in AGB in response to neighboring roots in the greenhouse experiment, and the modern variety showed less root proliferation in response to neighbors. We conclude that the newer cultivar was a weaker competitor but higher-yielding in two ways: (1) it had higher reproductive effort and therefore less allocation to structures that increase competitive ability, and (2) it had reduced root proliferation in response to the roots of neighboring plants. Our results show that wheat plants change their biomass allocation in response to resource levels and the presence of neighboring roots. The presence of root proliferation in the modern cultivar, albeit less than in the landrace, suggests that further increases in yield *via* group selection are possible.

## Introduction

Evolution in nature is driven by individual fitness, but the goal in agriculture is to optimize population performance (Denison et al., 2003; Weiner, 2003). Competition within a crop population reduces the performance of individual crop plants. Ideally, this reduction in individual production should be more than compensated by the production of neighboring individuals, so that population yield is maximized. But when there is competition among individuals, natural selection will favor “selfish” attributes and behaviors, which use resources to improve individual competitive performance at the expense of population performance (Zhang et al., 1999; Weiner et al., 2017), resulting in the so-called “tragedy of the commons” (Hardin, 1968; Rankin et al., 2007), which is undesirable in crop populations. Population yield will be highest in a single-species population (like most agricultural fields with weed control) if individual plants do not use resources competing with one another but cooperate when obtaining shared resources.

The most widely recognized “selfish” attribute of crops is being tall. Height growth consumes resources, and yield will be higher in a monoculture if all plants are short and allocate more to yield production. Breeding of shorter cultivars led to some of the significant increases in yield during the “green revolution” (Poehlman, 2013). Artificial selection for higher yields has resulted in shorter plants that allocate fewer resources to grow in height and more to reproduction (Specht and Williams, 1984). Similarly, selection for higher yields has reduced root size (Zhu et al., 2019), reducing the resource acquisition capacity of individual plants and weakening their competitiveness with neighbors. Over the course of breeding for high yields in the past 120 years, the size of crop roots has been reduced, root architecture has been simplified, and the overlap between roots of nearby plants has been reduced (Zhu et al., 2019).

In general, landraces, which are genetically close to their wild ancestors, have strong competitiveness, while recent cultivars, which have been artificially selected for increased population yield over many generations, are weaker competitors (Song et al., 2010; Weiner et al., 2017).

Some forms of phenotypic plasticity in response to neighbors are also “selfish” and detrimental to population yield (Weiner, 2004). One example is root proliferation in response to the presence of neighboring roots. Because below-ground resources often limit fitness, natural selection has resulted in plants that preferentially proliferate their roots in areas of the soil with high resource levels (Campbell and Grime, 1989; Murren et al., 2020), and this is also the case in agricultural fields (Nye and Tinker, 1977; Colom and Baucom, 2020). It has been hypothesized that some plants can sense the presence of the roots of neighboring individuals and proliferate roots as a pre-emptive competitive strategy (Zhang et al., 1999). Consistent with this hypothesis, there is evidence that some crop species [soybean (Gersani et al., 2001); Kenya beans (Maina et al., 2002); pea: (O’Brien et al., 2005)] exhibit an increase in root production and a decrease in seed yield in response to interplant competition below ground. Other species’ roots respond only to resource levels, thereby growing roots away from neighboring roots that have reduced soil resource levels [*Viola tricolor* (Lankinen, 2008), *Andropogon gerardii* (Markham and Halwas, 2011), Oat (Semchenko et al., 2007), and *Brassica rapa* (McNickle and Brown, 2014)]. Some species appear to follow a fixed root growth plan and simply grow slower when they experience lower resource levels due to competition for belowground resources (Mou et al., 2013).

Not only are there different strategies in response to root competition, but root growth is also influenced by environmental factors, such as rooting volume, soil resource levels, and interactions with other organisms such as mycorrhizae (Schenk, 2006; Hess and De Kroon, 2007; O’Brien et al., 2007; Novoplansky, 2009). This has led some researchers to question the evidence in support of root proliferation in response to neighboring roots (Chen et al., 2015, 2020, 2021).

“Selfish” attributes and behaviors increase individual fitness. They thereby may have contributed to increasing yields at the beginning of crop domestication as newly domesticated crops were selected for their performance in a managed agricultural environment. However, they now have negative effects, because they increase allocation to active competition with neighbors, thus reducing population performance. Therefore, it may be possible to increase yields by optimizing non-self root responses in crops (Denison et al., 2003).

Our main objectives here were to investigate the competitive performance of older and newer cultivars of wheat, and to ask if root proliferation in response to neighboring roots plays a role in competition and biomass allocation. We hypothesized that (a) biomass and its allocation reflect the group selection during crop breeding, such that recent higher-yielding crop cultivars show less “selfish” behaviors; (b) the crop plants with strong competitiveness have more root proliferation in response to the roots of neighboring plants, increasing the “tragedy of the commons.”

## Materials and Methods

### Field Experiment 1

The field experiment was conducted at the Experimental Station of Lanzhou University in Yuzhong County, Gansu Province, China (104°09’ E, 35°56’ N, altitude 1749 m) from March to July 2016. Eight hexaploid spring wheat cultivars released over the past hundred years were used in this experiment. These represent a sequence of locally bred cultivars with increasing yields (Table 1). The site is representative of a semi-arid climate within north-western China, with 30-year average precipitation of 168 mm, mean pan evaporation of 938 mm, mean temperature of 14^°^C, and mean relative humidity of 59% during the wheat growing season. The soil type is loessial soil with texture of silty loam.

**TABLE 1:** Name and source of cultivars used in the experiment.

Cultivar	Release time	Source
HST	Before 1949	Landrace
JBY	Before 1949	Landrace
GS96	1950s	GAAS
DX24	1963	DAAS
DX35	1979	DAAS
LC8139	1986	GAAS
LC8275	1997	GAAS
GC25	2008	GAAS

*Sources were landrace, the Gansu Academy of Agricultural (GAAS), the Dingxi Academy of Agricultural Science (DAAS).*

The experiment used a split-plot randomized complete block design. Two planting densities were set in main plots: 256 plants/m^2^ (the standard seeding rate for this region) and 128 plants/m^2^ (low planting density), in separate blocks 2 m apart. The eight cultivars were randomized within three replicates in subplots. Each plot measured 1.5 m × 1.5 m, and the spacing between neighboring plots was 0.5 m.

Following a basal dose of nitrogen (120 kg/ha), phosphorus (60 kg/ha), and potassium (48 kg/ha), wheat grains were sown at a depth of 4 cm in a uniform grid pattern by hand through a 1.5 m × 1.5 m frame with a grid of nylon wires forming the 24 × 24 grid pattern giving 256 grains/m^2^, and in alternate rows to obtain 128 grains/m^2^. A fungicide (triadimefon) and an insecticide (dimethoate) were sprayed as required to prevent disease and insect damage, and weeds were removed by hand.

At 121 days after sowing, we harvested a centrally placed 1 m^2^ subplot within each plot to determine aboveground biomass (AGB). Root samples were collected using a drill (10 cm diameter) from 0 to 1 m, washed carefully to remove soil, dried for 48 h at 80°C, and then weighed.

### Field Experiment 2

This field experiment was conducted from March to July 2013 at the Experimental Station of Lanzhou University in Yuzhong County, Gansu province, China (104°09’ E, 35°56’ N, altitude 1,749 m). The soil is a loess-like loam classified as an Orthic Entisol. The average bulk density was 1.29 g/cm^3^, the field water holding capacity (by weight) was 27.8%. The soil was plowed to make it homogeneous. A landrace (HST) and a modern cultivar (LC8275) were used in this experiment. These two cultivars have similar phenology, plant height, and shoot architecture and have been studied in recent literature (Song et al., 2009, 2010; Zhu et al., 2019, 2020; Du et al., 2020). The two cultivars are easy to distinguish in mixtures because LC has awns while HST does not.

There were three treatments with three replicates in a randomized complete block design: (a) HST monoculture, (b) LC monoculture, and (c) two cultivar mixtures in the ratio 1:1. Each plot measured 3 m × 3 m. Following a basal dose of nitrogen (120 kg/ha), phosphorus (60 kg/ha), and potassium (18 kg/ha), wheat was sown 4 cm deep in rows spaced 20 cm apart. The seeding rate was 250 grains/m^2^, which is the recommended rate in this region.

The number of plants and crop yield of all plots was measured by excavating two quadrats (5 rows × 1 m long × 10 cm deep) 122 days after sowing when the plants were mature. After carefully washing the roots, we separated the plants, counted the number of plants, number of tillers on each plant, and determined grain number and weight. All samples were dried for 48 h at 80^°^C and weighed, except grain yield, which was air-dried.

The competitiveness of two cultivars was calculated respectively by relative yield [RY; (Keddy et al., 1994)]:


RY=Y/mixYmono

where Y_mix_ and Y_mono_ are the dry matter yield of the wheat cultivar in mixture or monoculture, respectively. An RY > 0.5 means that the cultivar is a strong competitor, whereas RY < 0.5 indicates a weaker competitor, and RY = 0.5, means they have same competitiveness.

### Greenhouse Experiment

The two contrasting wheat cultivars, HST and LC, were also used in a greenhouse pot experiment. The experiment was carried out in a heated greenhouse (25°C/15°C, day/night temperature) at the Experimental Station from October 2012 to March 2013.

**FIGURE 1:**
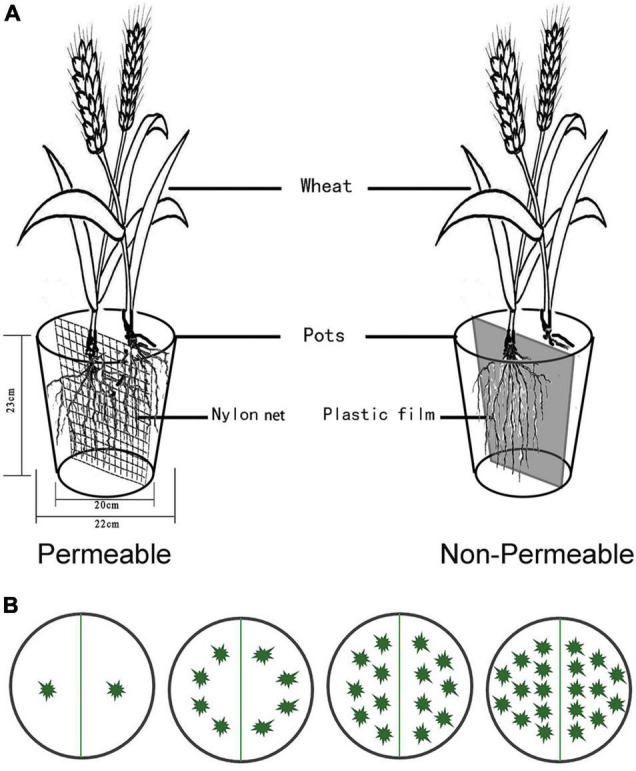
Schematic diagram of the experimental units. **(A)** The partition between the two halves of the pot. Permeable (segregated by nylon net, “net”) and Non-Permeable (segregated by plastic film, “film”). **(B)** The planting density and layout.

Plastic pots (diameter = 22 cm; height = 23 cm) were partitioned into two parts by nylon mesh (aperture size: 74 μm) or thick plastic (Figure 1A) to ensure the same soil volume in all treatments and avoid errors caused by difference of soil spaces. Each pot was filled with 5 kg of a 1: 3 (volume) mixture of vermiculite and a silt soil obtained from the top 20 cm of soil at the field experimental site and passed through a 10-mm sieve. The vermiculite was used to prevent soil compaction, facilitate root sampling, and an additional layer (100 g) of vermiculite was placed on the soil/vermiculite mixture to reduce soil evaporation. The field (pot) capacity (FC) of the mixture was 32.7% (w/w). Before sowing, 1 g N, 0.27 g P, and 0.35 g K per pot were applied in an aqueous solution of NH_4_NO_3_ and KH_2_PO_4_.

We used a multivariate randomized block design with four factors: (a) two types of root dividers (Figure 1A): Non-Permeable (plastic film, “film”) and Permeable (74 μm-nylon net, “net,” just allowed moisture and nutrients to pass through); (b) two watering regimes (90 and 40% FC, starting 30 days after sowing), with variation during the day < 5%; (c) three cultivar combinations (HST monoculture, LC monoculture, mixture between HST and LC); (d) four densities (2, 8, 14, 20 plants per a pot, Figure 1B). There were 3 pots for each monoculture treatment and 6 pots for each mixture treatment. In addition to the pots to be harvested at maturity, 72 pots were sown for harvesting 30, 60, and 90 days after sowing. These units consisted only of the mixture treatments with eight plants per pot under 90 and 40% FC with six replicates.

After sterilization with 75% alcohol, grains were vernalized at 4°C for 24 h. Wheat grains were placed on filter paper moistened with distilled water and placed in the dark to germinate at 25°C in an incubation cabinet. After radicles emerged, the germinated grains were sown according to planting density treatments (Figure 1B). After emergence, all plants were watered daily to maintain the soil water content of 90% FC (by weight) until the beginning of the water treatments. We added water to two cultivars mixture pots by a ratio of consumptions of two cultivars in monoculture at every time of keeping the moisture, to eliminate the experimental error caused by different water absorption capacity of two cultivars in the mixture.

Plants in the 72 pots for the 3 early samplings and the 192 pots harvested at maturity were separated into leaf, stems (including leaf sheaths), and spikes. Roots were separated from the soil by carefully washing with water until they were totally clean. All plant material was dried for 48 h at 80^°^C and weighed. We measured the shoot, root, and grain dry matter, and calculated reproductive effort [also called harvest index (HI) = grain biomass/total biomass, and root to shoot ratio (R/S)]. In addition, the relative change in biomass due to net vs. film dividers (RBD) was quantified as


RBD=[BY-(film)BY](net)/BY×(film)100%

where BY_(film)_ and BY_(net)_ are the biomass yield of the wheat cultivar in film or net dividers, respectively. A positive value means there was less biomass with net dividers, while a negative value means that plants benefitted from net rather than film dividers.

### Statistical Analyses

Root and shoot biomass were log-transformed to normalize and homogenize the variances. Pearson correlation analyses were conducted to test the correlation between biomass and release year of each cultivar in field experiment 1. All variables in field experiment 2 were tested for differences between cultivar combinations using analysis of variance (ANOVA). Only the differences of variables between non-Permeable and Permeable treatments at each level were tested by ANOVA based on our hypothesis. Mean comparisons were made by Fisher’s protected least significant difference (LSD) at *P* = 0.05 significance level. All statistical analyses were performed using GenStat (version 17; VSN International, Hemel Hempstead, United Kingdom). Statistical tests were considered significant if *p* ≤ 0.05.

## Results

### Biomass Allocation in the Field Experiment 1

**FIGURE 2:**
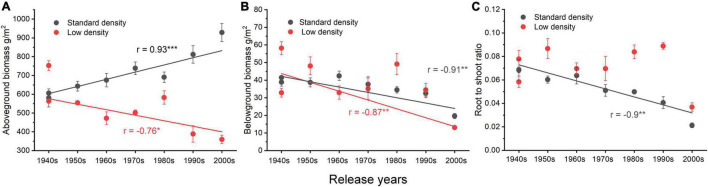
Relationships between biomass and release year of eight cultivars in two planting densities. **(A)** The relationship between aboveground biomass and release time, **(B)** the relationship between belowground biomass and release time, and **(C)** the relationship between the root to shoot ratio and release time. Error bars indicate standard errors. *p ≤ 0.05, **p ≤ 0.01, ***p ≤ 0.001.

The relationships between AGB and the release time of wheat cultivars showed very different trends at different planting densities. There was a significant positive correlation at the standard planting density (*r* = 0.93, *p* < 0.001) but a significant negative correlation at the low planting density (*r* = −0.76, *p* < 0.05, Figure 2A). Belowground biomass was negatively correlated with release time of cultivars at both planting densities (standard density: *r* = −0.91, *p* < 0.01; low density: *r* = −0.87, *p* < 0.01, Figure 2B). The R/S of cultivars at standard planting density decreased significantly with release date (*r* = −0.9, *p* < 0.01), but there was no correlation at low density (Figure 2C).

### Competitiveness of the Cultivars in the Field Experiment 2

**FIGURE 3:**
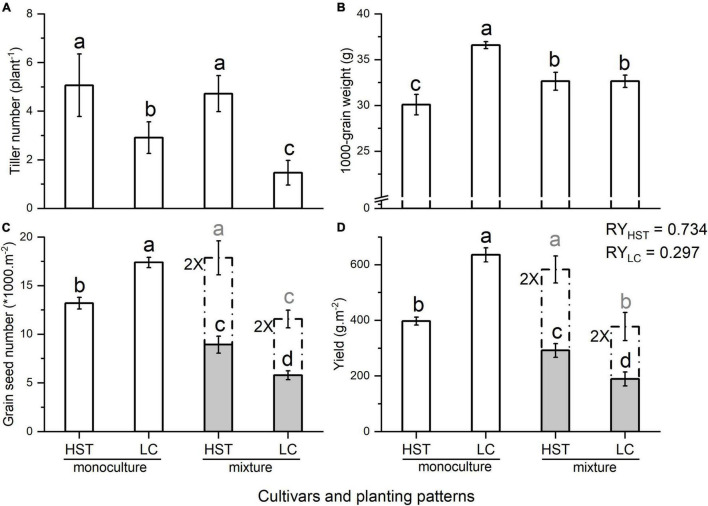
Yield components in Field Experiment 2. **(A)** Tiller number; **(B)** 1,000-grain weight; **(C)** Grain seed number; **(D)** Yield. Error bars indicate one standard error above and below the mean. HST and LC are tested cultivars, RY: relative yield. Dashed bars represent doubling of the population variables for comparison purposes.

Each cultivar had approximately 220 plants/m^2^ in the monoculture and 110 plants/m^2^ in mixture treatments. HST, the landrace, had the highest average number of tillers (5 plant^–1^) when grown with the modern cultivar LC. The tiller number of LC decreased from 3 plant^–1^ in monoculture to 1.47 plant^–1^ when growing with HST (Figure 3A). LC had the heaviest 1,000-grain weight in monoculture, which decreased significantly when in mixture with HST, but the 1,000-grain weight of HST increased significantly from 30.10 g in monoculture to 32.65 g in mixture (Figure 3B and Supplementary Table 1). LC produced significantly more and heavier grains than the HST in monoculture, but the pattern was reversed in mixture (Figures 3C,D and Supplementary Table 1). The relative yield of HST was 0.734, while LC’s relative yield was 0.297.

### Shoot and Root Biomass in the Greenhouse Experiment

**FIGURE 4:**
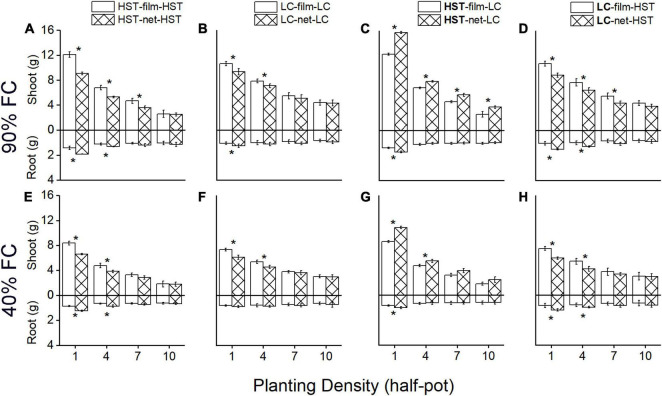
Mean root and shoot (stems plus leaves and spike) mass per plant of two cultivars in the pot experiment. Panels **(A–D)** are at 90% field capacity (FC), panels **(E–H)** are at 40% FC. Panels **(A,B,E,F)** are cultivars HST and LC in monoculture. Panels **(C,D,F,G)** are HST and LC in mixture. In the mixtures, the cultivar in bold is the target plant; soil dividers: “film”’ or “net.” All values are grams dry mass per individual. The error bars indicate one standard error above and below the mean. The asterisks indicate significant differences between two soil dividers.

In the monocultures (Figures 4A,B,E,F), the film divider treatment resulted in significantly more shoot and less root biomass than the net divider treatment at all water levels. Shoot biomass (stem plus leaves) per plant was on average 33% (*p* < 0.05) higher, and mean root mass per individual plant was 25.7% (*p* < 0.05) lower in the film than the net treatment for HST, and total mass increased on average by 23% (*p* < 0.05) when planting density was 1 plant/half-pot. LC showed the same response to the net vs. film dividers as HST, but to a lesser degree.

In mixtures (Figures 4C,D,G,H), the shoot, root, and total biomass were similar to the monocultures with film dividers, but there was a large difference with net dividers. The landrace HST had significantly more shoot and root biomass in the “net” than the “film” treatment. Per individual plant of landrace HST, shoot biomass (stem plus leaves) was on average 23.6% higher (*p* < 0.05), and mean root mass per individual plant was on average 23% higher (*p* < 0.05) in “net” than “film” treatment, and total mass increased on average by 28% (*p* < 0.05) when planting density was 1 plant/half-pot. The trend in biomass production of the modern cultivar LC was in the opposite direction than that of the landrace HST in inter-cultivar competition: the shoot and total biomass decreased on average by 17.3 and 11.6%, while root biomass increased on average by 46% more for “net” individuals than for “film” at 90% FC and 1 plant/half-pot.

Plant density and watering regimes significantly influenced shoot and total biomass in all treatments (Figure 4). Plants had less above-and belowground biomass in the low- than in the high-water treatment. The differences in shoot, root, and total biomass between “film” and “net” became weaker as the density increased. The largest total biomass of HST individuals occurred under “net” and 1 plant/half pot (Figure 4C) in inter-cultivar competition with LC; LC’s highest biomass occurred in “film” at the lowest density (Figure 4D).

**FIGURE 5:**
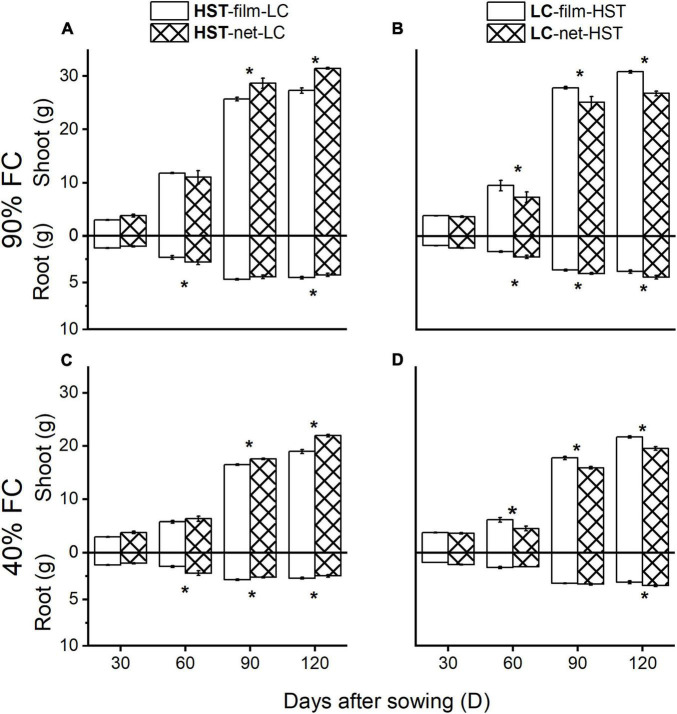
Average shoot and root biomass of two cultivars. Samples from in inter-cultivar competition and eight plants/pot treatment in plant growth stage. Panels **(A,B)** at 90% field capacity (FC) and panels **(C,D)** at 40%. Panels **(A,C)** are cultivar HST and panels **(B,D)** are LC. The target cultivar is in bold. Asterisks indicate significant differences between two soil dividers (*P* < 0.05).

**FIGURE 6:**
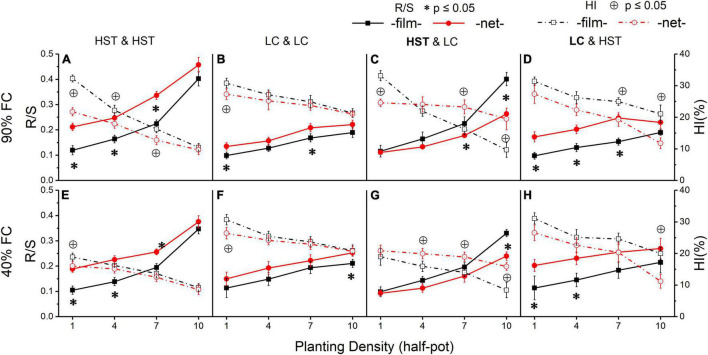
Root to shoot ratio (R/S) and harvest index (HI) of two cultivars grown in the pots. Panels **(A–D)** are at 90% FC, panels **(E–H)** are at 40% FC. Panels **(A,B,E,F)** are HST and LC in monoculture; panels **(C,D,F,G)** are HST and LC in mixture. The cultivar in bold is the target in inter-cultivars treatment; “film” means separated by film; “net” means separated by net. All values are based on g dry mass per individual. The error bars indicate one standard error R/S and HI the mean. * and ^⊕^ indicate significant differences in R/S and HI, respectively, between two soil divider treatments.

A significant (*p* < 0.05) effect on above- and belowground biomass due to root competition appeared after 60 days (Figure 5). The shoot biomass of the HST was significantly higher in the “net” than in the “film” treatment 90 DAS (Figure 5A), but shoot biomass of LC in “net” was lower than in the “film” treatment (Figure 5B). Nevertheless, the root biomass was higher in the “net” treatment than in the “film” treatment at 60 DAS. The differences between biomass for “net” and “film” were larger at 90 than 40% FC (Figure 6).

### Root:Shoot Ratio and Harvest Index

The experimental treatments significantly affected R/S. Overall, R/S increased with increasing density (Figure 6). Cultivar HST (Figures 6A,E) was more sensitive to density than LC (Figures 6B,F). In monoculture, the R/S of HST was significantly higher in the “net” than in the “film” treatment, but the R/S of LC showed a significant difference at only a few planting densities. R/S was significantly different in the two cultivars in mixture, and HST’s R/S was significantly higher in “film” than “net” at the high planting densities (7 or 10 plants/half-pot; Figure 6C). In contrast, R/S of cultivar LC was lower in “film” than “net” (Figures 6D,H) at low planting density (1 or 4 plants/half-pot).

There was a negative correlation between HI and planting density, with higher R/S values corresponding to lower HI. In monoculture, LC had higher HI than HST (Figures 6A,B,E,F). In mixture, the response of HI in LC was opposite to that of HST, in that HST had higher HI in the “net” than in “film” treatment (Figures 6C,G), but LC’s HI responded in the opposite direction (Figures 6D,H).

### Reduction in Aboveground Biomass Due to Net Dividers

**FIGURE 7:**
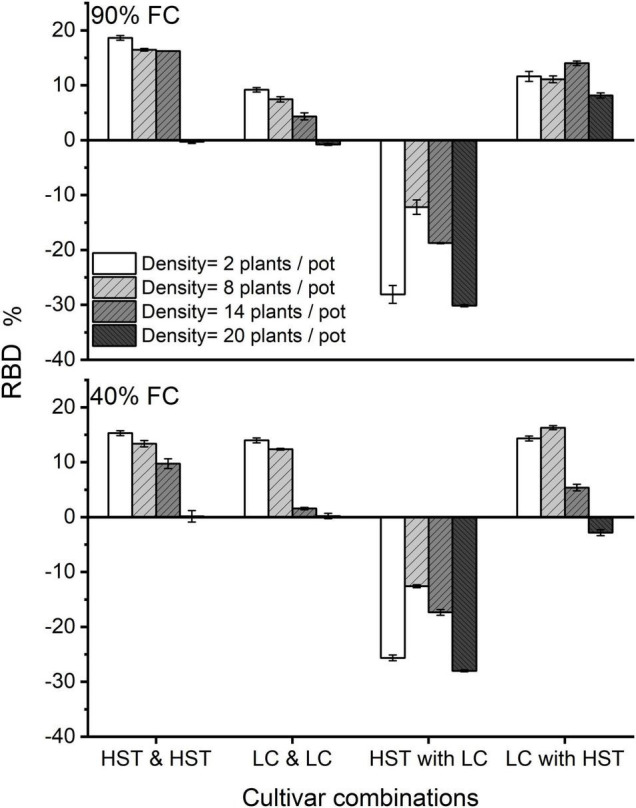
Effects of net vs. film dividers of two cultivars at different densities. RBD is the changes in root biomass due to net vs. film dividers. HST and LC are the cultivars. HST and HST/LC and LC signify the same cultivar on both sides of the dividers. HST with LC and LC with HST refers to different cultivars in a pot. FC is field water capacity. The error bars indicate one standard error of the mean.

Root-root interactions between the halves of the soil volume in the “net” treatment increased belowground and reduced aboveground biomass in monocultures of both cultivars, but to different degrees, and the effect was influenced by water level (Figure 7). Cultivar HST showed a greater reduction in AGB than LC in the monoculture at 90% FC, while the two cultivars had the same RBD in intra-cultivar competition at low density at 40% FC treatment. At the planting density of seven plants/half-pot, HST had greater RBD than LC in intra-cultivar competition, but the RBDs of both HST and LC were close to 0 when there were 10 plants/half-pot.

In the mixture, HST had negative RBD values, while LC had positive values when planting with HST. In mixtures with only 1 plant/half pot, HST had an RBD almost equal to the value in 10 plants/half-pot, and RBD of HST had decreased with planting density from 4 to 10 plants/half-pot. The watering regimes did not affect the RBD of either cultivar at low density.

## Discussion

### Biomass Allocation Reflects Evolutionary Selection

Biomass is the material foundation of grain yield and plant competitiveness, and numerous tradeoffs among attributes of resource acquisition organs have been documented. Therefore, total biomass and its allocation largely determine resource acquisition and plant competitiveness. For example, a larger root system can explore a larger soil volume to absorb more nutrients and water (Zhu et al., 2019), larger or more leaves intercept more light, and plant height determines the ability to shade neighbors and avoid being shaded. Biomass allocated to one structure is not available to other structures, and natural selection results in the tradeoffs that maximize individual fitness in nature.

Crop yield, on the other hand, is an attribute of the population. Improving individual competitiveness will reduce group performance (Weiner et al., 2017). The landrace allocated more dry matter to roots than did the modern cultivar (Figure 4), presumably due to natural selection for individual competitiveness, with the result that the landrace produced lower yield than the modern cultivar (Zhu et al., 2020). Over five decades of crop selection, newer varieties had lower root biomass and smaller root length density, but produced higher population yield than old cultivars (Aziz et al., 2017). We have previously found that the root:shoot ratio has been continuously reduced over the course of wheat breeding, and that root architecture has become simpler (Zhu et al., 2019).

Competition is density-dependent, almost by definition, so tradeoffs between individual and population performance will be affected by density. The AGBs in field experiment 1 showed different trends resulting from crop breeding at standard and low density (Figure 2A), indicating that competition among individuals of old varieties at standard density limited the performance of individuals, while the new varieties with high reproductive allocation cannot produce high yield at low density. We conclude that trends in biomass production and its allocation during crop cultivar evolution reflect the effects of group selection at high crop densities (Weiner et al., 2017).

The optimal population yield of a monoculture does not consist of strong competitors (Anten and Vermeulen, 2016). HST had a larger response to the presence of neighboring roots than LC in intra-cultivar competition (Figure 4). Thus, the newer cultivars show lower losses at high density and produce a high population yield than older more competitive cultivars.

In a study on 2 cultivars of maize, the advantage of the stronger competitor decreased with increasing planting density (Zhai et al., 2017). In our study, LC showed a reduction in shoot biomass due to increased density earlier than HST, which had the advantage in inter-cultivar competition (Figure 5). Thus, the new cultivar with weak competitiveness is more sensitive to neighbors. The new cultivar produces a high yield at the cost of individual competitiveness favored by natural selection.

### Changes in Biomass Allocation in Response to Neighbors

A plant’s performance in competition is not only influenced by its fixed traits, those of its competitor, and the environment in which competition occurs, but also the plant’s plastic responses to neighbors. Some forms of plasticity that improve individual fitness are “selfish” and result in decreased population yield (Mou et al., 2013; Weiner et al., 2017). Root proliferation in response to neighbors is one of these.

Several researchers have criticized “root divider” vs. “no divider” tests of root proliferation in response to neighbors, arguing that root interactions are confounded with rooting volume in such experiments (Hess and De Kroon, 2007; Chen et al., 2015, 2020, 2021). In the present study, we tried to eliminate the confounding effect of soil volume by dividing the soil volume with a nylon net rather than a “no divider” treatment (Figure 1). This can reduce interactions among roots in the rhizosphere (Chen et al., 2020; McNickle, 2020), which means that the effects we observed are conservative, strengthening any conclusions. We chose two wheat cultivars that had a large difference in yield, but two cultivars of the same species are still much more similar than different species in nature. This makes any differences more surprising.

### Resolving Negative Responses in Evolutionary Agriculture

There is a maximum value of biomass production of single crop species per unit of land (Weiner and Freckleton, 2010). New crop cultivars produce higher grain yield than old varieties primarily due to changes in allocation, not through increased biomass production (Figures 2, 6). The root and shoot biomass of individual plants of both cultivars decreased with planting density (Figure 4), but R/S increased (Figure 6), because plants allocate a greater proportion of their biomass to roots at higher agricultural densities (O’Brien et al., 2005; Song et al., 2010; Fang et al., 2011) although R/S often decreases in wild plants at very high densities (Wang et al., 2021). A previous study showed that root architectural traits of wheat cultivars over the past 100 years showed changes in root architecture resulting in smaller, simpler, and more economic root system architecture, which has resulted in more efficient resource acquisition at the population level at the expense of individual performance in competition (Zhu et al., 2019).

We should begin by recognizing that natural selection has already optimized much of the genome of the plant prior to domestication (Gustafson et al., 2009). Nonetheless, domestication and subsequent breeding of crops have resulted in an enormous improvement in crop productivity under agricultural conditions, in addition to improvements in their suitability for human use. For crop plant populations, the relationship of individual fitness and population yield is unimodal (Weiner et al., 2017), suggesting that the optimal population in agriculture should be comprised of cultivars near the peak of the unimodal model, not at maximal plant fitness, as in nature. The optimal population for resource-use efficiency is not evolutionarily stable, so it is not what we should expect to see in nature. Many opportunities for further improvements remain, so agronomists and ecologists should explore unselfish traits to develop “communal” cultivars to improve population yield.

Our results showed that root interactions resulted in a reduction in AGB in low density (Figure 7), and that this effect was stronger for the better competitor. As a result, groups that show restraint in competition over a common resource will produce higher yields than groups in which individual-level competition results in a “tragedy of the commons.” This is evidence that evolution of wheat cultivars has reduced individual crop plant competitiveness to increase population grain yield (Donald, 1968; Zhang et al., 1999).

## Conclusion

Competition for resources is important in agricultural fields as well as in nature. Natural selection maximizes individual fitness, but agriculture is about population performance. Therefore, the optimal strategy for crop production will be very different from that which natural selection produces. Wheat plants change their biomass allocation in response to resource levels and the presence of neighboring roots, and some of these responses reduce population performance. Plants that have strong competitiveness below ground allocate more biomass to roots as a competitive strategy. Such plants will have an advantage as individuals in competition but will also produce lower yields at the population level. Thus, if weeds are not abundant, the optimal crop plant will be a weak competitor, which does not use resources to compete actively with its neighbors but reacts to neighbors in the same way it reacts to its own ramets.

The modern cultivar showed less root proliferation in response to the roots of neighboring plants. Our results support the hypothesis that much of the increase in yields from plant breeding has been due to inadvertent group selection, which has reduced “selfish” behaviors and the resultant “tragedy of the commons.” The presence of root proliferation in the modern cultivar, albeit less than in the landrace, suggests that further increases in yield *via* group selection are possible.

## Data Availability Statement

The datasets presented in this study can be found in online repositories. The names of the repository/repositories and accession number(s) can be found below: https://figshare.com/s/1d480d55e3dbdf882061.

## Author Contributions

Y-HZ, YJ, and M-XY carried out experimental work. Y-HZ and JW performed the data analyses and wrote the manuscript. F-ML initiated the study, contributed to the design of experiments and analysis of the data, and assisted with writing the manuscript. All authors contributed to the article and approved the submitted version.

## Conflict of Interest

The authors declare that the research was conducted in the absence of any commercial or financial relationships that could be construed as a potential conflict of interest.

## Publisher’s Note

All claims expressed in this article are solely those of the authors and do not necessarily represent those of their affiliated organizations, or those of the publisher, the editors and the reviewers. Any product that may be evaluated in this article, or claim that may be made by its manufacturer, is not guaranteed or endorsed by the publisher.
