# Supplementary material for: Biomass Allocation Responses to Root Interactions in Wheat Cultivars Support Predictions of Crop Evolutionary Ecology Theory
Source: Front Plant Sci. 2022 Mar 23;13:858636. doi: 10.3389/fpls.2022.858636 (PMC8984106; doi:10.3389/fpls.2022.858636)
Supplement: Supplementary file 1 [file Table_1.DOCX]

| Variable | | Source of variation | DF | | Sum of Squares | F Value | P Value |
| --- | --- | --- | --- | --- | --- | --- | --- |
| Plant number | Cultivar | | 1 | 14.083 | | 0.129 | 0.729 |
|  | Monoculture vs. mixture | | 1 | 2.083 | | 0.019 | 0.894 |
|  | Interaction | | 1 | 0.750 | | 0.007 | 0.936 |
|  | Error | | 8 | 876.00 | |  |  |
|  | Total | | 11 | 892.92 | |  |  |
| Tiller number | Cultivar | | 1 | 21.870 | | 126.064 | <0.001 |
|  | Monoculture vs. mixture | | 1 | 2.394 | | 13.800 | 0.006 |
|  | Interaction | | 1 | 0.908 | | 5.231 | 0.051 |
|  | Error | | 8 | 1.39 | |  |  |
|  | Total | | 11 | 26.56 | |  |  |
| 1000-grain weight | Cultivar | | 1 | 31.363 | | 29.061 | 0.001 |
|  | Monoculture vs. mixture | | 1 | 1.456 | | 1.349 | 0.279 |
|  | Interaction | | 1 | 31.623 | | 29.301 | 0.001 |
|  | Error | | 8 | 8.63 | |  |  |
|  | Total | | 11 | 73.08 | |  |  |
| Grain seed number | Cultivar | | 1 | 0.822 | | 0.604 | 0.459 |
|  | Monoculture vs. mixture | | 1 | 189.131 | | 139.030 | <0.001 |
|  | Interaction | | 1 | 40.407 | | 29.703 | 0.001 |
|  | Error | | 8 | 10.88 | |  |  |
|  | Total | | 11 | 241.24 | |  |  |
| Yield | Cultivar | | 1 | 13909.425 | | 27.984 | 0.001 |
|  | Monoculture vs. mixture | | 1 | 229597.368 | | 461.914 | <0.001 |
|  | Interaction | | 1 | 87663.160 | | 176.364 | <0.001 |
|  | Error | | 8 | 3976.46 | |  |  |
|  | Total | | 11 | 335146.41 | |  |  |

Zhu et al. (2022) Biomass allocation responses to root interactions in wheat cultivars support predictions of crop evolutionary ecology theory.

Table S1. Analysis of variance table for variables in field experiment 2.
